# Correction: Pronounced electronic modulation of geometrically-regulated metalloenediyne cyclization

**DOI:** 10.1039/d5sc90147b

**Published:** 2025-07-10

**Authors:** Sarah E. Lindahl, Erin M. Metzger, Chun-Hsing Chen, Maren Pink, Jeffrey M. Zaleski

**Affiliations:** a Department of Chemistry, Indiana University Bloomington IN 47405 USA zaleski@iu.edu; b Molecular Structure Center, Indiana University Bloomington IN 47405 USA

## Abstract

Correction for ‘Pronounced electronic modulation of geometrically-regulated metalloenediyne cyclization’ by Sarah E. Lindahl *et al.*, *Chem. Sci.*, 2025, **16**, 255–279, https://doi.org/10.1039/D4SC05396F.

The authors regret that the experimental values of Δ*G*^‡^ for the Bergman cyclization kinetics in their published work are incorrect due to a unit conversion error. Table 2 and Fig. 4, 7, and 8 are hereby corrected to the values as given below. Table 18S of the ESI has also been corrected. Despite the unit conversion error, all experimental trends observed in the cyclization kinetics are accurately reported in the original publication.

**Table 2:** Kinetic activation parameters for the thermal Bergman cyclization of complexes **3a–3e** (aryl) and **3f–3g** (alkyl) at 25 °C. Functionalization of aromatic rings with electron donating (**3b**, **3d**) and electron withdrawing groups (**3c**, **3e**) leads to acceleration and retardation of cyclization kinetics, respectively

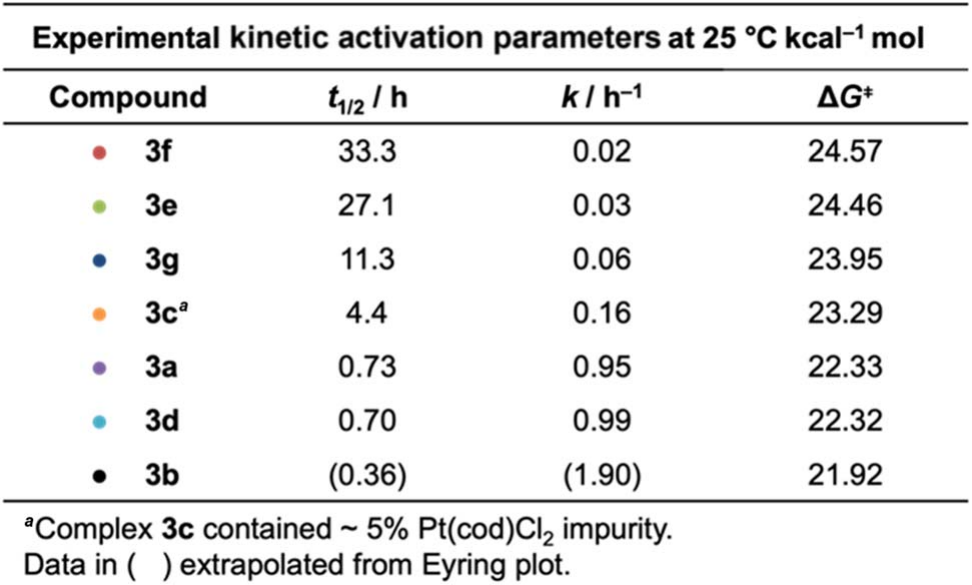

The published computational studies were performed with the pure density functional BPW91, which was chosen based on its ability to accurately model the experimental activation barriers to Bergman cyclization. After correcting the unit conversion error in the experimental Δ*G*^‡^ values, the BPW91 functional now underestimates the activation barriers to Bergman cyclization by 5–6 kcal mol^−1^; however, all computational trends originally reported are still consistent with the corrected experimental data.

After correcting the experimental unit conversion error, the B3PW91 functional accurately models activation barriers to within <2 kcal mol^−1^. Therefore, we have elected to update Fig. 4, 7, and 8 to include the B3PW91 data. All trends reported in these figures with the updated B3PW91 data are consistent with those originally published with BPW91 computed structures. Table 18S has also been added to summarize the B3PW91 computational results simply for completeness.

**Fig. 4:**
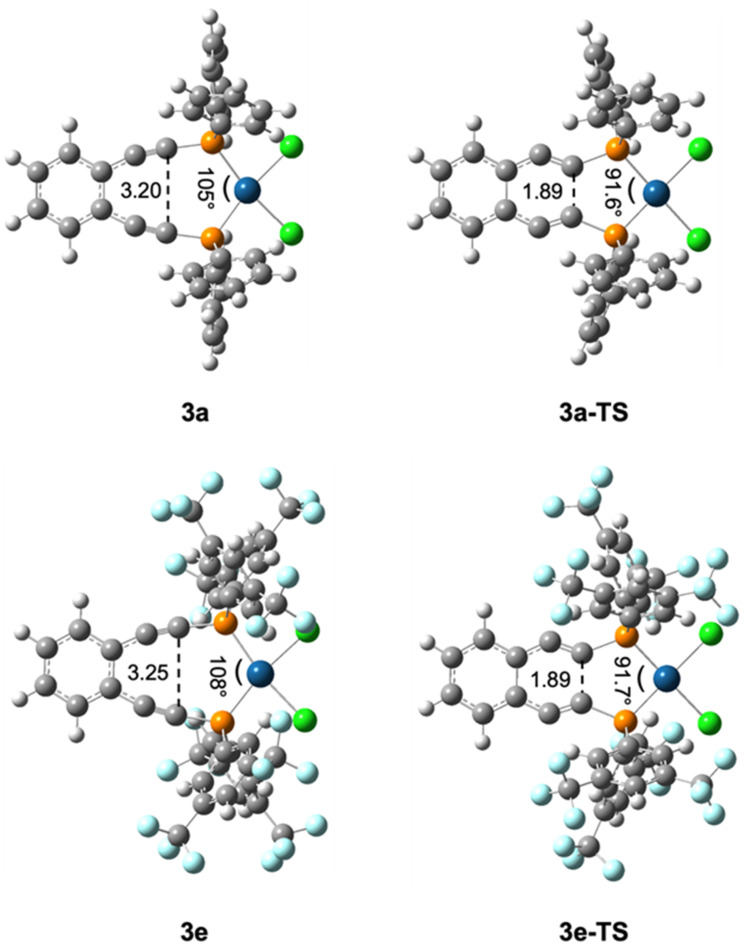
Key structural parameters of (U)B3PW91/6-31G**/LANL2DZ optimized metalloenediynes **3a** and **3e** and their transition states **3a-TS** and **3e-TS**. These bond length (Å) and angle (°) changes are representative of those observed for all metalloenediynes **3a–3e** and transition states **3a-TS–3e-TS**

**Fig. 7:**
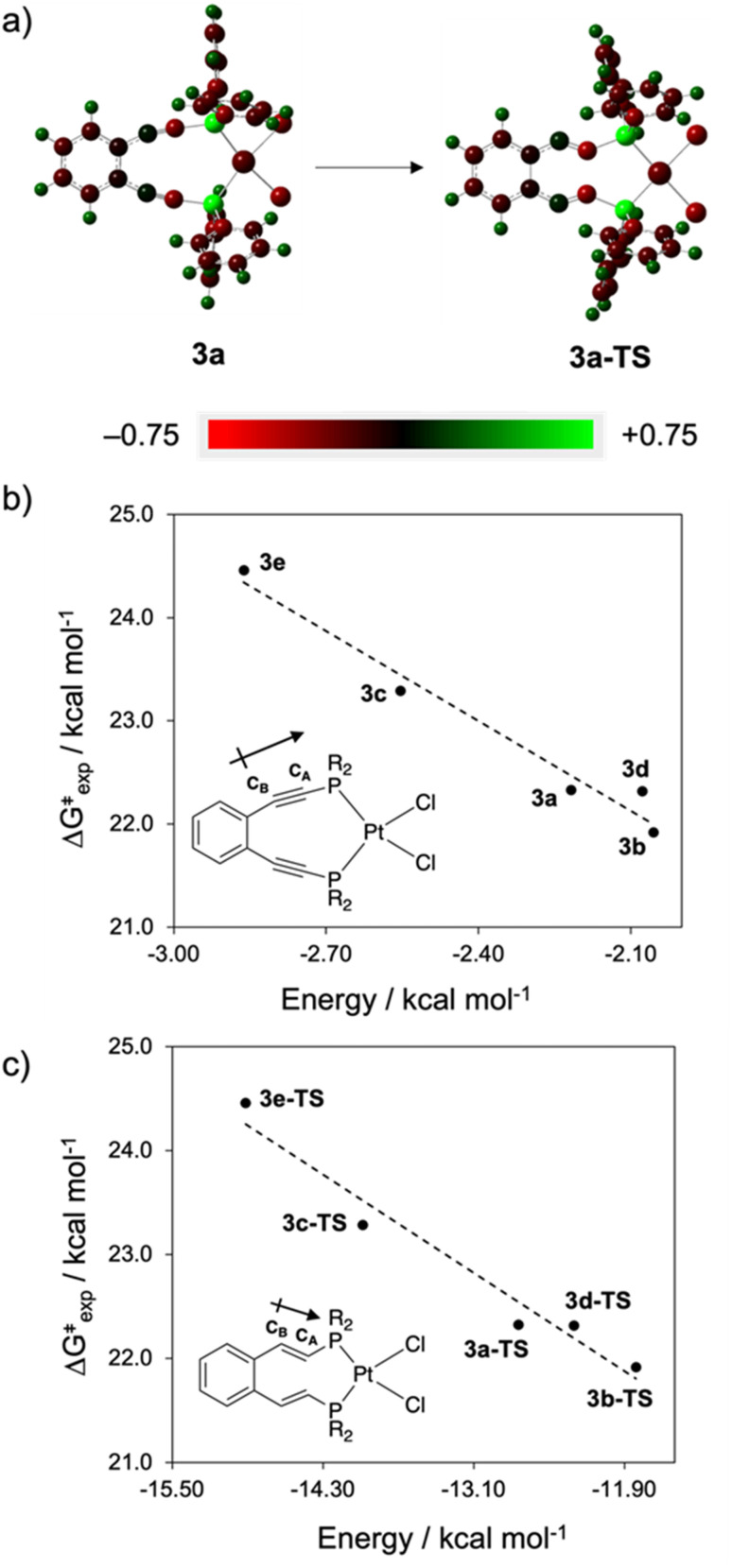
Plots of Δ*G*^‡^ as a function of alkyne dipole interaction energy, evaluated using (a) NBO charge analysis (representative example of **3a** shown) for (b) enediyne ground states (**3a–3e**) and (c) calculated transition states (**3a-TS–3e-TS**) demonstrating the relationship between alkyne polarity (C_A_–C_B_) and experimental barrier height to thermal Bergman cyclization. ((U)B3PW91/6-31G**/LANL2DZ).

**Fig. 8:**
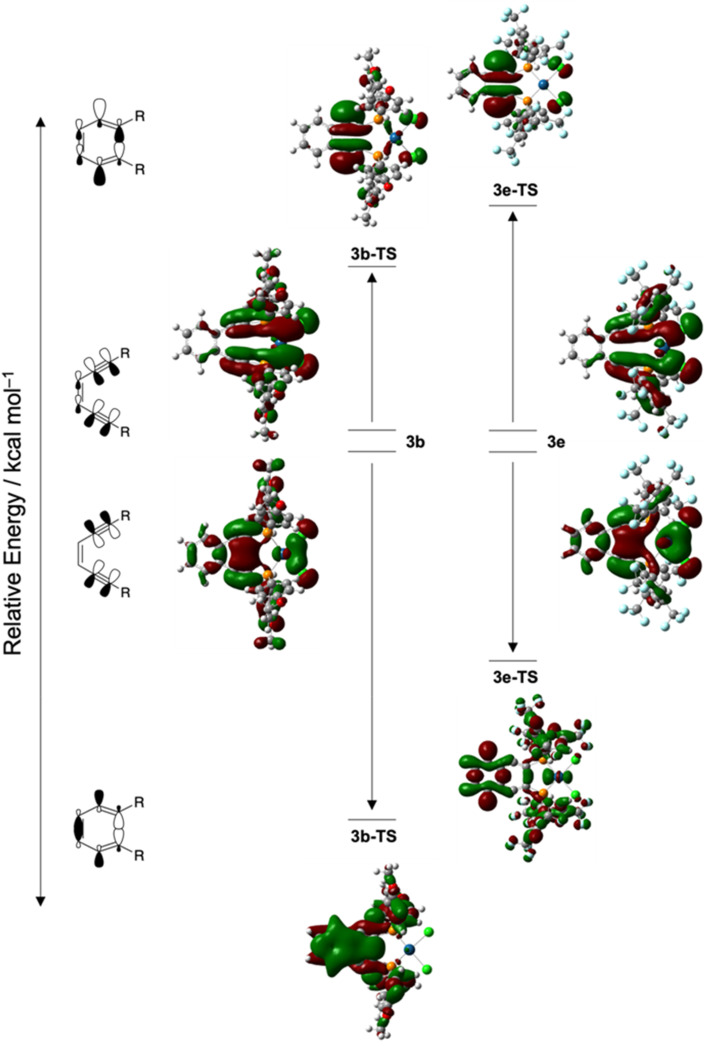
Walsh diagram showing the in-plane alkyne π/π*-orbitals most stabilized and destabilized by the cyclization reaction of complexes **3b** and **3e** and their associated transition states **3b-TS** and **3e-TS**. The degree of stabilization or destabilization of these orbitals in the transition state varies significantly due to the presence of electron donating (**3b**: R = Ph-*p*OCH_3_) or electron withdrawing (**3e**: R = Ph-*m*_2_CF_3_) groups at the alkyne termini. ((U)B3PW91/6-31G**/LANL2DZ).

The Royal Society of Chemistry apologises for these errors and any consequent inconvenience to authors and readers.

